# Study-level age patterns in cardiometabolic response to high-intensity interval training among adults with overweight or obesity: a systematic review and meta-analysis of randomized controlled trials

**DOI:** 10.3389/fphys.2026.1833719

**Published:** 2026-08-12

**Authors:** Ping Lin, Gang Xu, Xia Li

**Affiliations:** 1 School of Physical Education, Taiyuan University of Science and Technology, Taiyuan, China; 2 School of Physical Education, Sichuan University of Science & Engineering, Zigong, China; 3 School of Physical Education, Shanxi Datong University, Datong, China

**Keywords:** adults, age, cardiometabolic response, overweight or obesity, systematic review

## Abstract

**Background:**

HIIT is a time-efficient exercise strategy for adults with overweight or obesity, but trial findings are heterogeneous and whether age independently modifies cardiometabolic response remains uncertain.

**Methods:**

PubMed, Embase, the Cochrane Library, and Web of Science were searched from inception to December 2025. Random-effects meta-analyses estimated weighted mean differences for SBP and DBP in mmHg and standardized mean differences for lipid and anthropometric outcomes, with 95% confidence intervals and prediction intervals where available. Additional Stata analyses included funnel plots, Egger tests for all six outcomes, trim-and-fill analysis for BW and BMI, exploratory study-level meta-regression using continuous mean age and intervention duration, and GRADE assessment.

**Results:**

Nineteen trials involving 570 participants were included. HIIT was associated with reductions in SBP, DBP, TG, BW, and BMI and an increase in HDL-C; overall heterogeneity was moderate to substantial (I² range, 46.77%–87.07%). Egger tests suggested small-study effects for BW and BMI, but not SBP, DBP, HDL-C and TG. Trim-and-fill imputed no studies for BW and BMI. Mean age was associated with TG effects and duration was associated with HDL-C effects in univariable models. Certainty of evidence was low for SBP and very low for the remaining outcomes.

**Conclusion:**

HIIT was associated with favorable average cardiometabolic changes in adults with overweight or obesity, but substantial heterogeneity, low-to-very-low certainty and possible small-study effects findings require cautious interpretation. The available evidence supports the hypothesis that trial-level mean age may be associated with differential responses, rather than proving age as a definitive individual-level effect modifier.

**Systematic review registration:**

https://www.crd.york.ac.uk/prospero/, identifier CRD420261279689.

## 1 Introduction

Obesity remains one of the most urgent global public health challenges. Recent global surveillance indicates that in 2022 approximately one in eight people worldwide were living with obesity, and adult obesity has more than doubled since 1990, with parallel increases in adolescents (World Health Organization, [[NoYear]]a; World Health Organization, [[NoYear]]b). The health burden attributable to high body mass index (BMI) continues to rise, contributing substantially to preventable disability and premature mortality at the population level (Zhou et al., 2024). These trends are reinforced by policy focused reports that highlight insufficient national capacity and implementation gaps in obesity prevention and management strategies across many countries (World Obesity Federation, [[NoYear]]).

In addition to excessive body weight (BW) itself, obesity is also associated with various cardiometabolic abnormalities, including increased blood pressure, dyslipidemia, impaired glucose regulation and chronic low-degree inflammation, which together increase the risk of atherosclerotic cardiovascular disease and type 2 diabetes (Liu and Yang, 2025; Młynarska et al., 2025). Therefore, contemporary clinical guidelines emphasize that continuous lifestyle intervention should be the basis for obesity management and recommend planned physical activities, which not only helps weight management, but also helps to improve cardiometabolic risk indicators that affect long-term prognosis (Bae et al., 2025).

Against this background, high-intensity interval training (HIIT) has attracted attention as a time-efficient exercise strategy. Interval training alternates repeated high-intensity exercise bouts with lower-intensity recovery or rest, and HIIT is a structured form in which the high-intensity bouts provide the principal training stimulus (Coates et al., 2023). Compared with moderate-intensity continuous training, HIIT may improve cardiorespiratory fitness and selected metabolic outcomes with a lower total time commitment, which may be relevant for adults with obesity who commonly report limited time and low exercise tolerance as barriers to participation (Ko et al., 2025). Nevertheless, high exercise intensity may also increase acute cardiovascular demand and musculoskeletal loading. Screening, individualized progression, supervision, and adverse-event monitoring may therefore be particularly important for older adults with obesity (Costache et al., 2024).

Research evidence on HIIT in adults with overweight or obesity has expanded, but earlier syntheses answer somewhat different questions. A meta-analysis in metabolic syndrome pooled a broader high-risk population rather than adults selected specifically for elevated adiposity (Poon et al., 2024). Comparisons of HIIT with moderate-intensity continuous training have focused mainly on body composition and insulin sensitivity and found that changes in BW and BMI are generally modest and often similar between training modes (Sanca-Valeriano et al., 2023). A further review of people with obesity and diabetes considered a wider clinical phenotype and a broad set of cardiometabolic outcomes (Al-Mhanna et al., 2025). Furthermore, a recent meta-analysis specifically targeting obese adults confirmed that aerobic exercise significantly reduces both systolic and diastolic blood pressure, but primarily examined moderate-to-vigorous continuous aerobic protocols rather than interval-based training (Mandal et al., 2025). This leaves the specific effects of HIIT, and particularly study-level age-related variation in response, insufficiently characterized in adults with overweight or obesity. Overall, these research results support HIIT as a promising intervention strategy, but also emphasize that its effects vary across populations, interventions, and comparator conditions.

Despite the accumulation of existing evidence, there are still some important limitations. First of all, previous reviews have often combined overweight and obesity or pooled populations with different cardiometabolic diseases, potentially obscuring response patterns specific to adults with obesity (Rugbeer et al., 2021). Outcome reporting is also inconsistent, including mixtures of endpoint and change scores and differences in measurement protocols, which complicate cross-trial comparisons (Mathur, 2024). In addition, intervention duration and weekly frequency have been examined more often than age as potential sources of heterogeneity (Edwards et al., 2023). Exercise trials also frequently report intensity anchors, progression, adherence, and control conditions incompletely, limiting replication and evidence synthesis (Subialka et al., 2025; Wollesen et al., 2025).

Therefore, the aim of this study was to systematically review and meta-analyze RCT evidence on the effects of HIIT on clinically relevant cardiometabolic risk markers in adults with overweight or obesity. This study focused on six outcomes commonly used in cardiometabolic risk assessment: systolic blood pressure (SBP), diastolic blood pressure (DBP), high-density lipoprotein cholesterol (HDL-C), triglycerides (TG), BW, and BMI. In addition to estimating the overall pooled effect, this study explored whether between-trial heterogeneity was associated with study-level mean age, intervention duration, and weekly training frequency. The 55-year threshold was selected pragmatically to separate younger from middle-aged or older study samples and to approximate the upper range of the menopausal transition, during which cardiometabolic risk may accelerate. This rationale applies most directly to female-predominant samples and should be interpreted cautiously in mixed-sex trials; the threshold is not a universal biological cut-point. Because individual-participant data were unavailable, age analyses were ecological, study-level analyses and cannot determine whether age independently modifies individual responses. To aid clinical interpretation, a reduction of approximately 5 mmHg in SBP or DBP was considered a conservative benchmark for clinically relevant blood pressure improvement. By providing an updated synthesis and transparently evaluating heterogeneity, this review aims to inform exercise-prescription research while avoiding overinterpretation of subgroup findings.

## 2 Materials and methods

### 2.1 Protocol

This systematic review and meta-analysis was conducted and reported in accordance with the Preferred Reporting Items for Systematic Review and Meta-Analysis (PRISMA 2020) statement (Page et al., 2021). The methodological procedures were followed by Cochrane Handbook for systematic reviews of interventions (Jpt, 2008). The review protocol was prospectively registered in the International Prospective Register of Systematic Reviews (PROSPERO with registration No. CRD420261279689) to enhance transparency and minimize the risk of selective reporting (Booth et al., 2011).

### 2.2 Search strategy and study selection

A comprehensive literature search was independently performed by two reviewers in four electronic databases (PubMed, Embase, Cochrane Library, and Web of Science) from database inception to December 2025. Search strategies combined controlled vocabulary (MeSH/Emtree where applicable) with free-text keywords relating to high-intensity interval training, obesity, adults, and randomized trials. The Web of Science search strategy was:

TS=(“high intensity interval training” OR “high-intensity interval training” OR HIIT OR “high intensity intermittent training” OR “high-intensity intermittent training” OR “sprint interval training” OR “sprint interval exercise” OR SIT OR “interval training” OR “intermittent training” OR “interval exercise”) AND TS=(obesity OR obese OR “morbid obesity” OR adiposity OR “body mass index” OR BMI OR overweight) AND TS=(adult OR adults OR men OR women) AND TS=(randomized controlled trials OR Randomized controlled trials OR RCT).

All retrieved records were exported to Endnote software (version 21, Thompson ISI ResearchSoft) for citation management and duplicate removal. Two researchers independently screened the title and abstract of the literature, and then evaluated the qualification of the full text. Disagreements should be resolved through discussion, and the third researcher should be consulted if necessary. The complete screening process has been recorded in the PRISMA flowchart. In addition, the list of references that meet the criteria for inclusion in the standard research was manually screened to identify potential related tests that could not be retrieved by the database.

### 2.3 Inclusion and exclusion criteria

The qualification criteria for the inclusion of meta-analysis studies are determined by evaluating key elements, including the characteristics of the research subject, the type of intervention, the control conditions, the outcome indicators and the research design. The inclusion criteria include:

#### 2.3.1 Population

Adults (≥18 years) with obesity or overweight as defined by study authors (commonly BMI-based criteria). Studies from regions using population-specific BMI thresholds for obesity, including Asian criteria where applicable, were considered eligible, and the obesity definition used in each study was recorded. Because the eligibility criteria included both overweight and obesity, the population should be interpreted as adults with overweight or obesity rather than exclusively adults with obesity.

#### 2.3.2 Intervention

HIIT, including aerobic HIIT and sprint interval training (SIT), delivered as the primary exercise intervention. HIIT was defined as repeated bouts of high-intensity exercise interspersed with recovery periods or low-intensity intervals. Interventions of any exercise modality and implementation setting (supervised or unsupervised) were eligible.

#### 2.3.3 Comparator

Standard care or usual practice without a structured HIIT program. These terms reflected the original trial descriptions and could include routine clinical follow-up, general lifestyle advice, wait-list control, or maintenance of habitual activity. The available reports did not consistently specify whether participants undertook non-HIIT exercise, received dietary advice, changed medication, or altered background care; therefore, comparator variation was documented where possible and treated as a potential source of clinical heterogeneity and confounding.

#### 2.3.4 Outcomes

Trials reporting at least one prespecified outcome with extractable quantitative data at baseline and post-intervention (or change values). Primary outcomes were obesity-related cardiometabolic risk markers, including SBP, DBP, HDL-C and TG. Secondary outcomes were anthropometric indicators, including BW, and BMI.

#### 2.3.5 Study design

RCTs only.

If the research meets the following conditions, it is excluded: (1) Non-human research; (2) Participants were not adults with overweight or obesity; (3) HIIT/SIT was not the main intervention; (4) Combined co-interventions were used and the independent effect of HIIT could not be isolated; (5) extractable outcome data were unavailable; Or (6) non-randomized research designs, research protocols, editorials, reviews or conference abstracts without complete data. No restrictions were applied based on geographic region.

### 2.4 Data collection and management

Two researchers independently extracted data using a standardized and pilot-tested extraction table. The extracted information included: (1) publication information (first author, year of publication, country, and research design); (2) participant characteristics (sample size, mean age, sex distribution where reported, obesity or overweight definition, baseline BMI, and baseline cardiometabolic status where available); (3) intervention characteristics (exercise modality, intensity prescription, interval structure, session duration, weekly frequency, total intervention duration, progression, supervision, and adherence where reported); (4) comparator information, including usual-care content and background exercise, diet, or medication advice where reported; and (5) outcome data (baseline and post-intervention means and SDs, or mean changes and SDs). Extracted study characteristics were summarized descriptively and used to support exploration of heterogeneity.

For the multi-arm test, in order to avoid repeated counting, the treatment of the control group includes merging the relevant intervention groups or splitting the sample size of the control group if necessary. When the outcome indicators are reported in different units, such as blood lipid indicators, they should be uniformly converted into consistent units of measurement before the evidence is synthesized. When SD is not directly reported, the standard statistical conversion method is used to deduce according to the standard error, confidence interval or p value. Any disagreements in extraction were resolved by consensus, with third-reviewer adjudication if required. When necessary, outcome values reported graphically were extracted using digitization methods to enable inclusion in quantitative synthesis.

### 2.5 Quality appraisal

**Figure 1:**
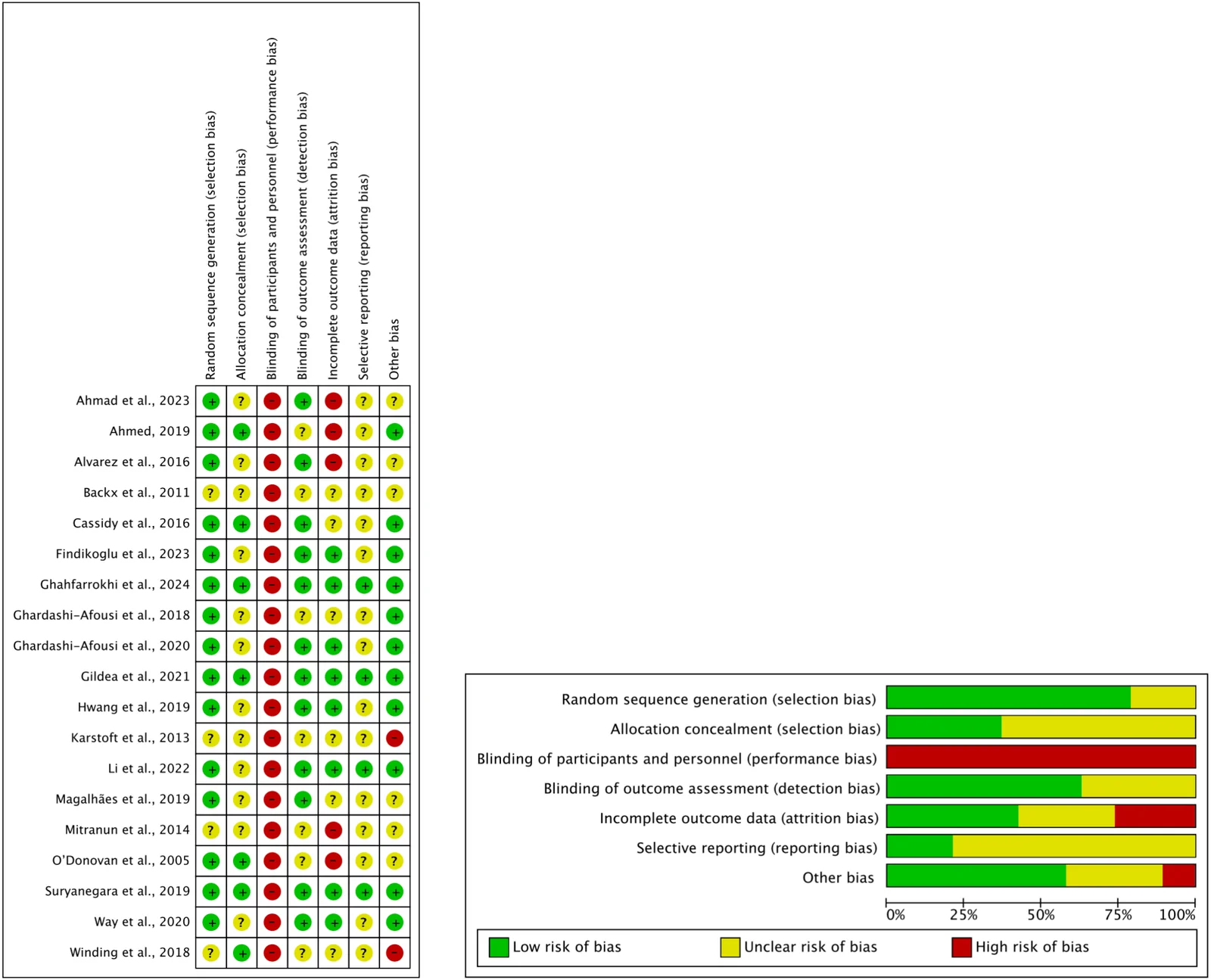
Risk of bias assessment of included RCTs (Cochrane RoB tool): traffic-light plot and summary.

The bias risk included in RCT is evaluated by two researchers independently using the Cochrane Randomized Trial Bias Risk Assessment Tool (ROB 1), covering seven areas: random sequence generation, distribution concealment, research subject and researcher blinding, outcome evaluation blinding, incomplete outcome data, selective reports and other potential bias (Higgins et al., 2011). RoB 1 was retained because it was the prespecified framework used to complete the original assessment and generate **Figure 1**. Each domain was judged as low, unclear, or high risk of bias. Discrepancies were resolved by discussion or third-reviewer adjudication. Risk-of-bias summary and traffic-light plots were produced using Review Manager (RevMan 5.4) (Cochrane Collaboration, 2020).

### 2.6 Statistical analysis

All analysis is carried out using Stata (version 18). The continuity outcome indicators (HDL-C, TG, BW and BMI) are combined with standardized mean difference (SMDs), SBP and DBP are combined with weighted mean differences (mmHg), and their 95% confidence intervals (CIs) and crediction intervals (CIs). Evidence certainty for each outcome was assessed using GRADE across risk of bias, inconsistency, indirectness, imprecision, and publication bias; RCT evidence started at high certainty and was downgraded when concerns were serious. Statistical heterogeneity is evaluated by Cochran’s Q test and quantified by I² statistics (Higgins et al., 2003). The interpretation of heterogeneity combined statistical indicators with clinical and methodological differences between studies, including participant characteristics, HIIT protocol features, comparator type, intervention duration, supervision, and outcome measurement procedures (Egger et al., 1997).

Random-effects models were used as the main analytical approach because clinical and methodological heterogeneity was expected. The DerSimonian-Laird estimator was retained for consistency with the prespecified analysis, but results were interpreted conservatively because conventional random-effects methods can provide over-precise inference when the number of studies is small and heterogeneity is substantial (DerSimonian and Laird, 1986). Fixed-effect estimates were examined only as sensitivity checks when heterogeneity was low and were not used to override the primary random-effects interpretation. Age was prespecified as a potential source of heterogeneity rather than assumed to be a definitive individual-level effect modifier. Subgroup analyses stratified studies by trial-level mean age (>=55 years vs. <55 years), intervention duration (12 weeks vs. non-12 weeks), and weekly training frequency (3 sessions/week vs. non-3 sessions/week). The 55-year threshold was chosen pragmatically to distinguish middle-aged or older samples from younger adult samples and to approximate the upper range of the menopausal transition, but this rationale is most applicable to female-predominant studies and should be applied cautiously to mixed-sex samples (Hulteen et al., 2023; Uddenberg et al., 2024).

Subgroup differences were evaluated using interaction tests, consistent with meta-analytic guidance (Borenstein et al., 2021). Exploratory random-effects meta-regression examined continuous study-level mean age and intervention duration separately and jointly. Additional covariates suggested by clinical reasoning, including sex proportion, baseline BMI, intervention modality, supervision, comparator content, and baseline cardiometabolic status, were considered but could not be modeled reliably across outcomes because reporting was incomplete and the number of trials per outcome was limited. Therefore, all subgroup and meta-regression findings were treated as hypothesis-generating study-level evidence vulnerable to ecological bias and residual confounding. Leave-one-out sensitivity analyses were used to identify influential studies, and funnel plots, Egger tests, and trim-and-fill analyses were used to assess small-study effects where appropriate.

## 3 Results

### 3.1 Search results and study characteristics

**Figure 2:**
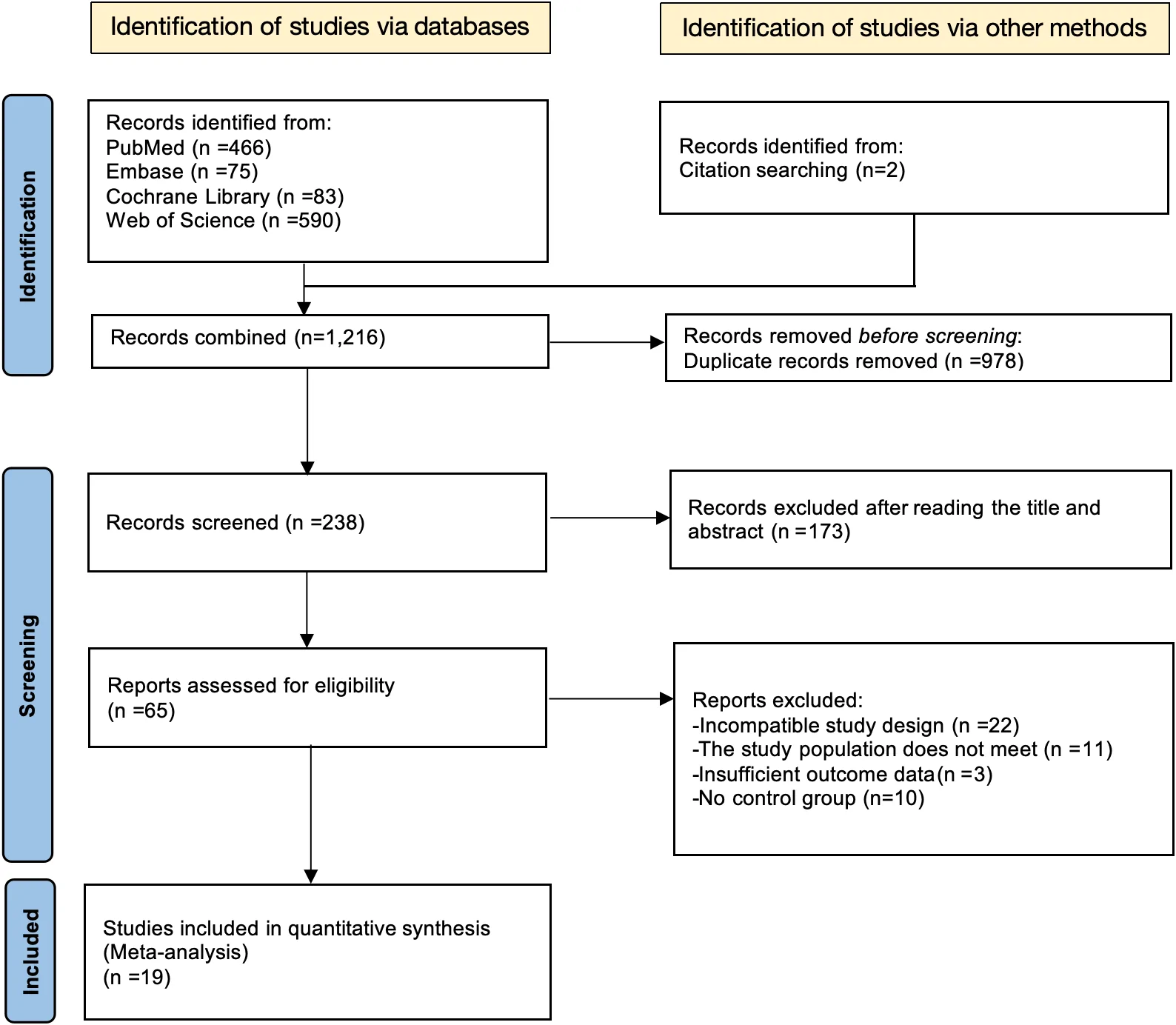
PRISMA flow diagram of study selection.

The database search identified 1,214 records from PubMed (n=466), Embase (n=75), the Cochrane Library (n=83), and Web of Science (n=590), and an additional two records were identified through citation searching. After combining records (n=1,216), 978 duplicates were removed, leaving 238 records for title/abstract screening. Of these, 173 were excluded, and 65 full-text reports were assessed for eligibility. Following full-text review, 46 reports were excluded due to ineligible study design (n=22), ineligible population (n=11), no control group (n=10), or insufficient outcome data (n=3). Ultimately, 19 RCTs were included in the quantitative synthesis (meta-analysis) (**Figure 2**). All included studies were published in English and were RCTs published up to December 2025.

The 19 included trials enrolled 570 participants (HIIT n=291; control n=279) and were conducted across multiple regions, most commonly in Europe, particularly the United Kingdom (5 studies), followed by Iran (3 studies), Denmark (2 studies), and Egypt (2 studies). Single studies were conducted in Thailand, Chile, the United States, Portugal, Australia, China, and Turkey (**Supplementary Table 1**).

Overall, participants were middle-aged to older adults, with mean ages ranging from 39.0 ± 5.0 to 67.5 ± 5.8 years across studies. Most interventions lasted 12 weeks (13/19 studies), with durations ranging from 6 to 24 weeks. Training frequency was typically 3 sessions/week (16/19 studies), with fewer studies prescribing 4 sessions/week (1/19) or 5 sessions/week (2/19). Given the wide age range across included trials, study-level mean age was prespecified as a potential source of heterogeneity; however, these aggregate data cannot substitute for individual-participant age-stratified analyses.

HIIT protocols varied in interval structure and intensity prescription (e.g., %HRmax, %HRR/HRpeak, RPE, or %VO_2_peak) and were most often implemented using cycling and/or walking/jogging/running, delivered in supervised (laboratory-based) or field-based formats depending on the trial (**Supplementary Table 1**). Control groups received routine care or usual practice without structured HIIT. The content of usual care, including exercise, diet, and medication management, was inconsistently described, preventing a more detailed between-study comparison.

### 3.2 Quality evaluation

The risk of bias assessment across the 19 included RCTs is shown in **Figure 1**. Low risk ratings were observed in 15 studies for random sequence generation, 7 for allocation concealment, 12 for blinding of outcome assessment, 8 for incomplete outcome data, 4 for selective reporting, and 11 for other bias, corresponding to 78.9%, 36.8%, 63.2%, 42.1%, 21.1%, and 57.9%, respectively. Unclear risk ratings were most common for allocation concealment, 12 of 19 studies, 63.2%, and selective reporting, 15 of 19 studies, 78.9%, mainly because methodological details, trial protocols, or prespecified analysis plans were often unavailable. High risk ratings were most prominent for blinding of participants and personnel, which was rated as high risk in all studies, 19 of 19, 100.0%, reflecting the nature of exercise-based interventions. High risk was also identified for incomplete outcome data in 5 studies, 26.3%, and other bias in 2 studies, 10.5%. Overall, the main methodological concerns were performance bias, unclear reporting of allocation concealment and selective reporting, and attrition bias in some trials.

### 3.3 Meta-analysis outcomes

#### 3.3.1 Blood pressure response to HIIT (SBP and DBP)

**Figure 3:**
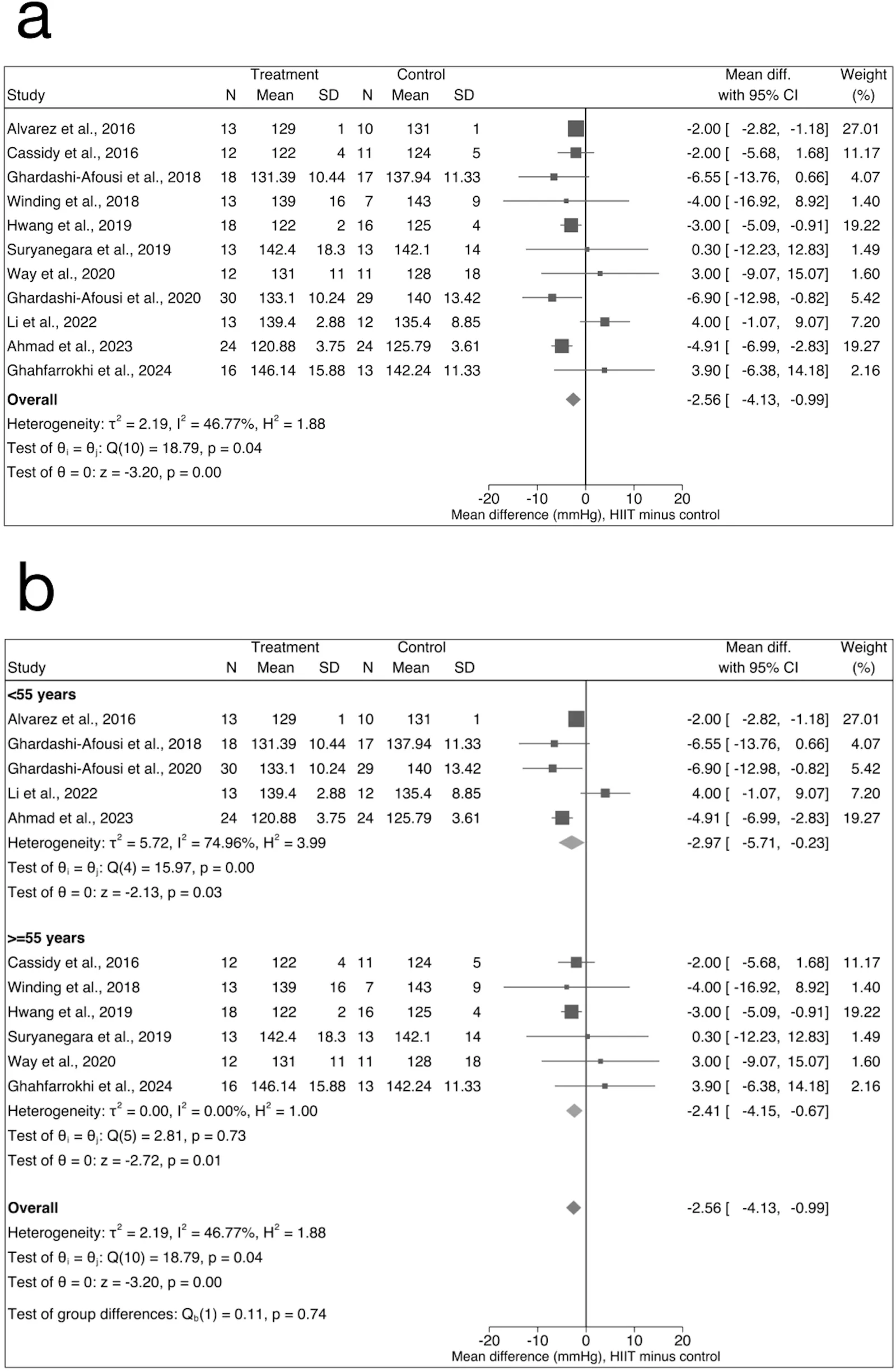
Effect of HIIT on SBP: **(a)** overall meta-analysis and **(b)** subgroup analysis by age (≥55 vs. <55 years).

For SBP, 11 RCTs were included in the weighted mean difference analysis using a random effects model. Compared with control conditions, HIIT was associated with a statistically significant reduction in SBP, with a pooled WMD of −2.56 mmHg, 95% CI −4.13 to −0.99, 95%PI -6.626 to 1.504. Moderate heterogeneity was observed across studies, I² = 46.77%, Q(10) = 18.79, p = 0.04 (**Figure 3a**). In the prespecified age subgroup analysis, HIIT was associated with significant SBP reductions in both age groups. Among participants younger than 55 years, the pooled WMD was −2.97 mmHg, 95% CI −5.71 to −0.23, with substantial heterogeneity, I² = 74.96%. Among participants aged 55 years and older, the pooled WMD was −2.41 mmHg, 95% CI −4.15 to −0.67, with no observed heterogeneity, I² = 0.00% (**Figure 3b**). The test for subgroup differences was not statistically significant, Qb(1) = 0.11, p = 0.74, indicating that the magnitude of SBP reduction did not differ significantly between age groups.

**Figure 4:**
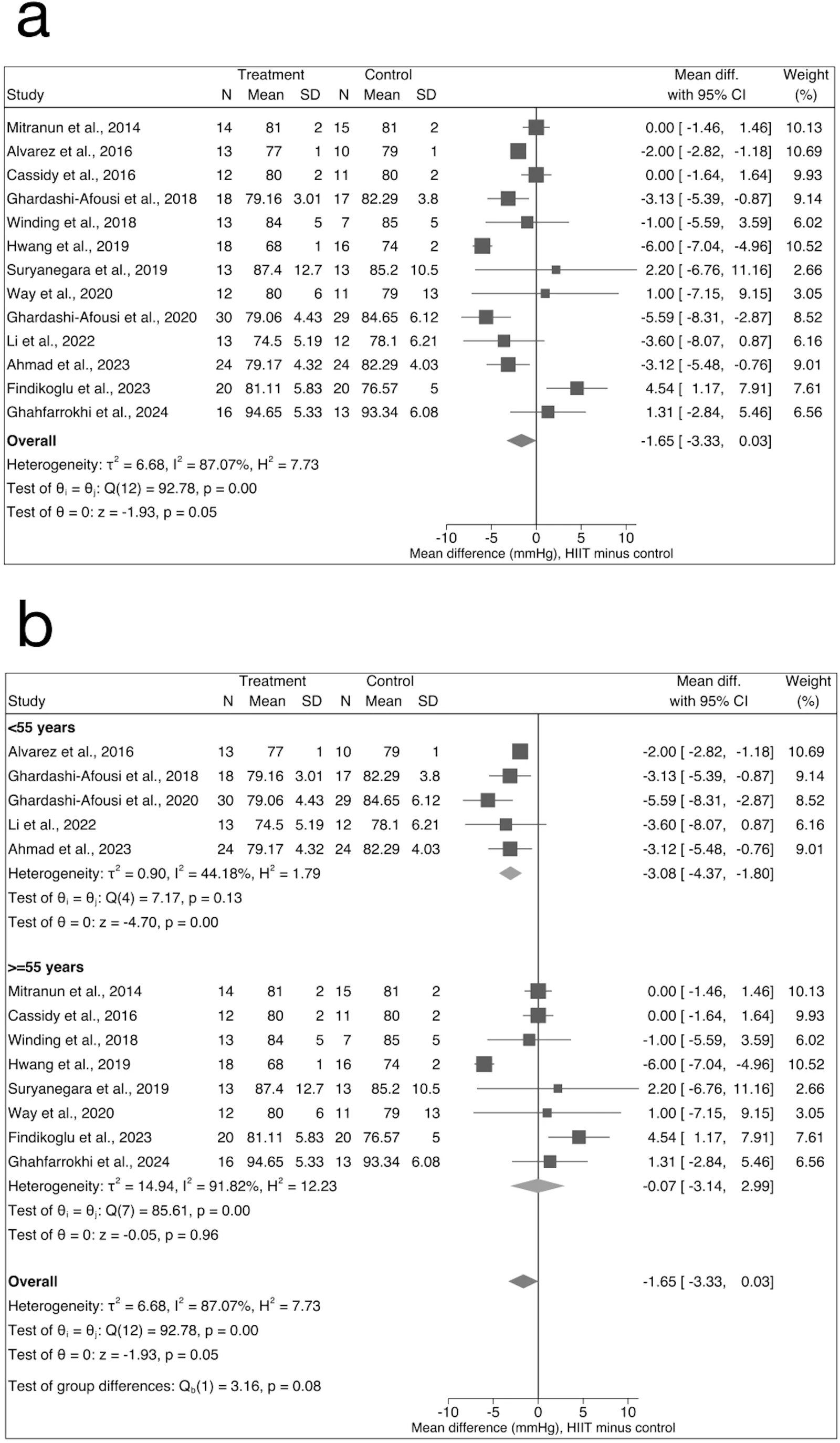
Effect of HIIT on DBP: **(a)** overall meta-analysis and **(b)** subgroup analysis by age (≥55 vs. <55 years).

For DBP, 13 RCTs were included in the weighted mean difference analysis using a random effects model. Compared with control conditions, HIIT was associated with a borderline reduction in DBP, with a pooled WMD of −1.65 mmHg, 95% CI −3.33 to 0.03, 95% PI -7.824 to 4.546. Considerable heterogeneity was observed across studies, I² = 87.07%, Q(12) = 92.78, p < 0.001 (**Figure 4a**). In the prespecified age subgroup analysis, HIIT was associated with a significant reduction in DBP among participants younger than 55 years, with a pooled WMD of −3.08 mmHg, 95% CI −4.37 to −1.80, and moderate heterogeneity, I² = 44.18%. In contrast, the pooled effect among participants aged 55 years and older was not statistically significant, WMD = −0.07 mmHg, 95% CI −3.14 to 2.99, with substantial heterogeneity, I² = 91.82% (**Figure 4b**). The test for subgroup differences did not reach statistical significance, Qb(1) = 3.16, p = 0.08, indicating that the apparent age related difference in DBP response should be interpreted cautiously.

Supplementary subgroup analyses by intervention duration (12 weeks vs non–12 weeks) and weekly frequency (3 sessions/week vs non–3 sessions/week) were performed for SBP and DBP; however, these analyses did not meaningfully reduce heterogeneity, indicating that residual variability may not be adequately explained by these program characteristics alone.

#### 3.3.2 TG response to HIIT: exploratory study-level age and duration patterns

**Figure 5:**
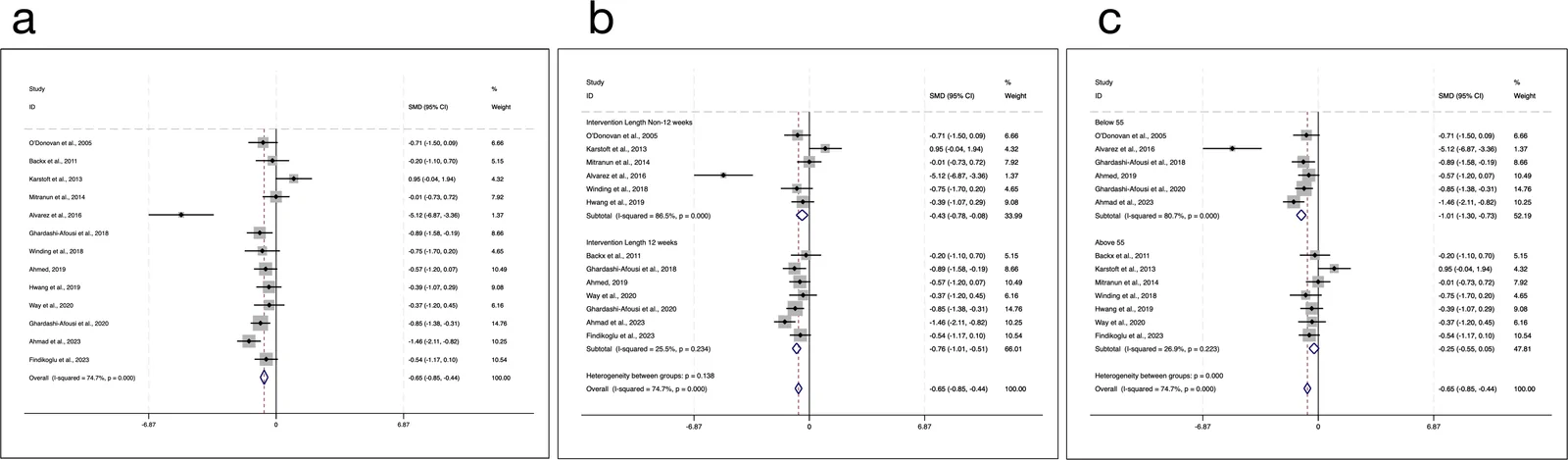
Effect of HIIT on TG: **(a)** overall meta-analysis, **(b)** subgroup analysis by intervention duration (12 weeks vs non–12 weeks), and **(c)** subgroup analysis by age (≥55 vs. <55 years).

A total of 13 RCTs reported TG outcomes (**Figure 5a**). The pooled meta-analysis showed that HIIT significantly reduced TG compared with controls (SMD = −0.65, 95% CI −0.85 to −0.44, 95% PI -2.709, 1.283), with substantial heterogeneity (I² = 74.7%, p < 0.001).

Subgroup analyses indicated that intervention duration and study-level mean age were informative but not definitive. In the duration subgroup analysis (**Figure 5b**), trials with a 12-week intervention showed a clear TG reduction (SMD = -0.76, 95% CI -1.01 to -0.51) with low-to-moderate heterogeneity (I^2^ = 25.5%), whereas non-12-week interventions also showed a significant but smaller effect (SMD = -0.43, 95% CI -0.78 to -0.08) with high heterogeneity (I^2^ = 86.5%). In the age subgroup analysis (**Figure 5c**), HIIT was associated with a larger TG reduction in trials with mean participant age <55 years (SMD = -1.01, 95% CI -1.30 to -0.73), but heterogeneity remained high (I^2^ = 80.7%). In trials with mean participant age >=55 years, the pooled effect was not statistically significant (SMD = -0.25, 95% CI -0.55 to 0.05) and heterogeneity was lower (I^2^ = 26.9%). The between-subgroup difference was significant (p < 0.001), suggesting that trial-level mean age may be associated with TG response. Because these analyses used study-level mean age rather than individual-participant data, they remain vulnerable to ecological bias and confounding by other trial-level differences. Subgrouping by weekly training frequency did not meaningfully reduce heterogeneity.

#### 3.3.3 Effect of HIIT on HDL-C: significant overall improvement without a clear age interaction

**Figure 6:**
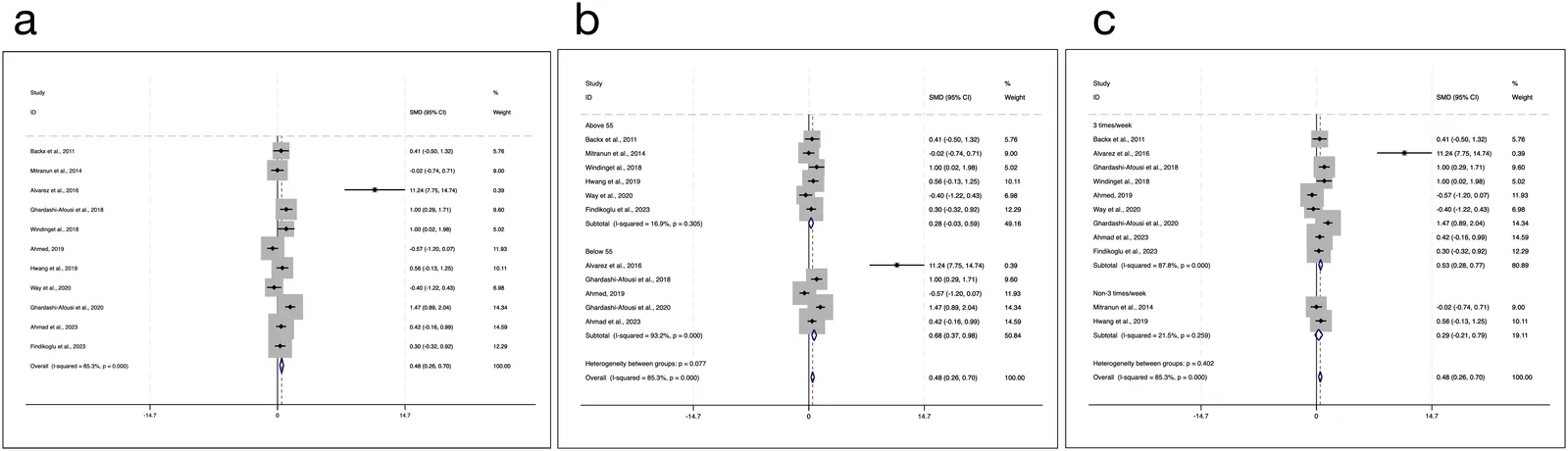
Effect of HIIT on HDL-C: **(a)** overall meta-analysis, **(b)** subgroup analysis by age (≥55 vs. <55 years), and **(c)** subgroup analysis by weekly frequency (3 sessions/week vs non–3 sessions/week).

As shown in **Figure 6a**, HIIT significantly increased HDL-C compared with controls (SMD = 0.48, 95% CI 0.26 to 0.70, 95% PI -4.111 to 6.201), with substantial heterogeneity (I² = 85.3%, p < 0.001).

In the age subgroup analysis (**Figure 6b**), HDL-C improved significantly in participants <55 years (SMD = 0.68, 95% CI 0.37 to 0.98; I² = 93.2%), whereas the effect was not statistically significant among those ≥55 years (SMD = 0.28, 95% CI −0.03 to 0.59; I² = 16.9%). However, the between-subgroup difference did not reach statistical significance (p = 0.077), indicating that study-level age grouping did not provide clear statistical evidence of age modification and did not fully account for heterogeneity.

For weekly frequency (**Figure 6c**), trials prescribing 3 sessions/week showed a significant increase in HDL-C (SMD = 0.53, 95% CI 0.28 to 0.77; I² = 87.8%), while studies with non–3 sessions/week did not demonstrate a significant effect (SMD = 0.29, 95% CI −0.21 to 0.79; I² = 21.5%). The between-subgroup difference was not significant (p = 0.402). Subgrouping by intervention duration did not meaningfully reduce heterogeneity, suggesting that remaining inconsistency may reflect differences in protocol design and participant characteristics. Although trials prescribing 3 sessions/week showed significant within-subgroup improvements in HDL-C, the between-subgroup difference was not significant (p = 0.402), providing no statistical evidence that weekly frequency modified the HDL-C response.

#### 3.3.4 Anthropometric outcomes: modest reductions in BW and BMI with persistent heterogeneity

**Figure 7:**
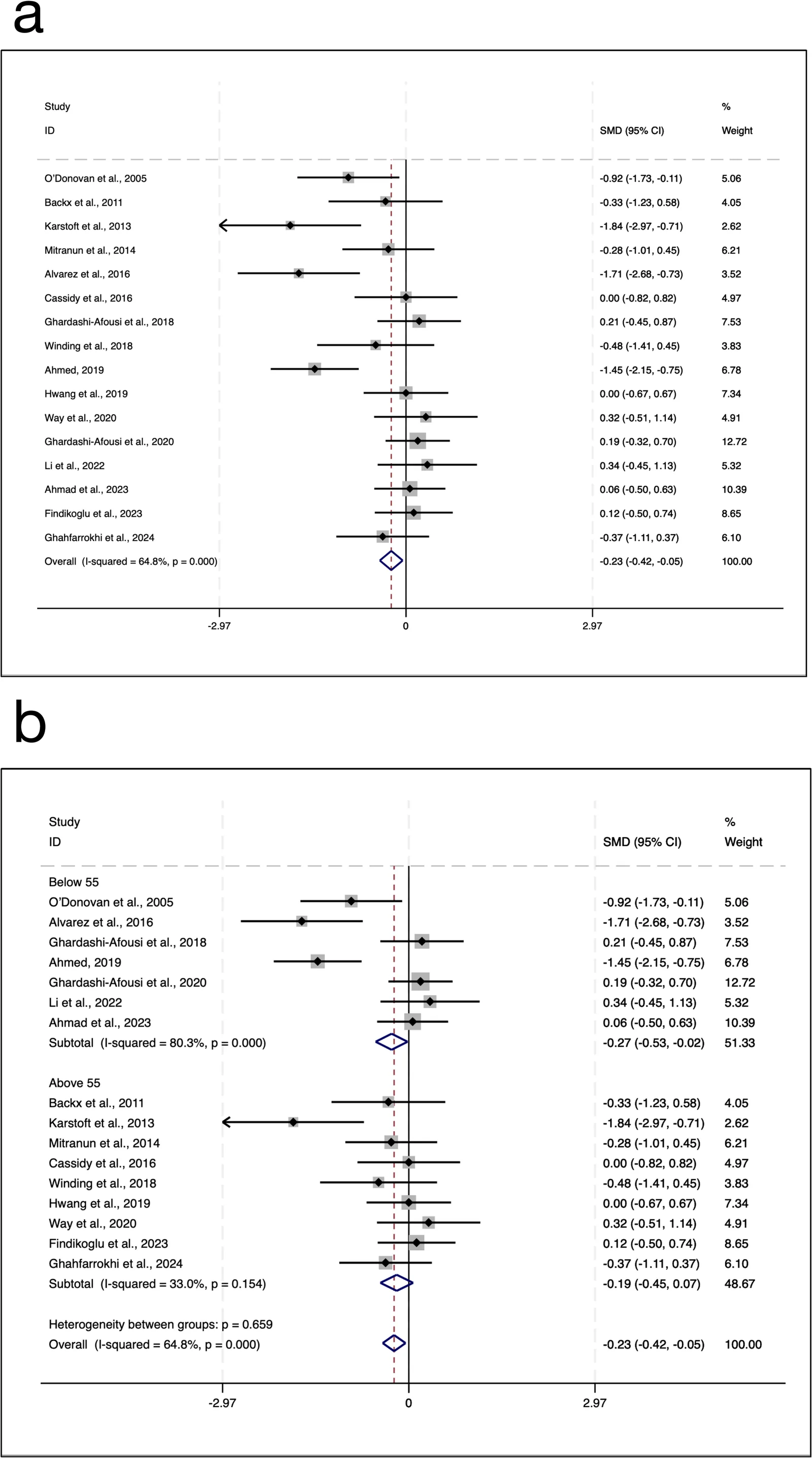
Effect of HIIT on BW: **(a)** overall meta-analysis and **(b)** subgroup analysis by age (≥55 vs. <55 years).

For BW, a total of 16 RCTs reported BW outcomes (**Figure 7a**). HIIT was associated with a small but statistically significant reduction in BW compared with controls (SMD = −0.23, 95% CI −0.42 to −0.05, 95%PI -1.511 to 0.861), with moderate-to-substantial heterogeneity (I² = 64.8%, p < 0.001). In the age subgroup analysis (**Figure 7b**), HIIT was associated with a small significant BW reduction among participants <55 years (SMD = −0.27, 95% CI −0.53 to −0.02), although heterogeneity remained high (I² = 80.3%). Among participants ≥55 years, the effect was not statistically significant (SMD = −0.19, 95% CI −0.45 to 0.07) and heterogeneity was lower (I² = 33.0%). The between-subgroup difference was not significant (p = 0.659), suggesting no clear age modification for BW in this dataset.

**Figure 8:**
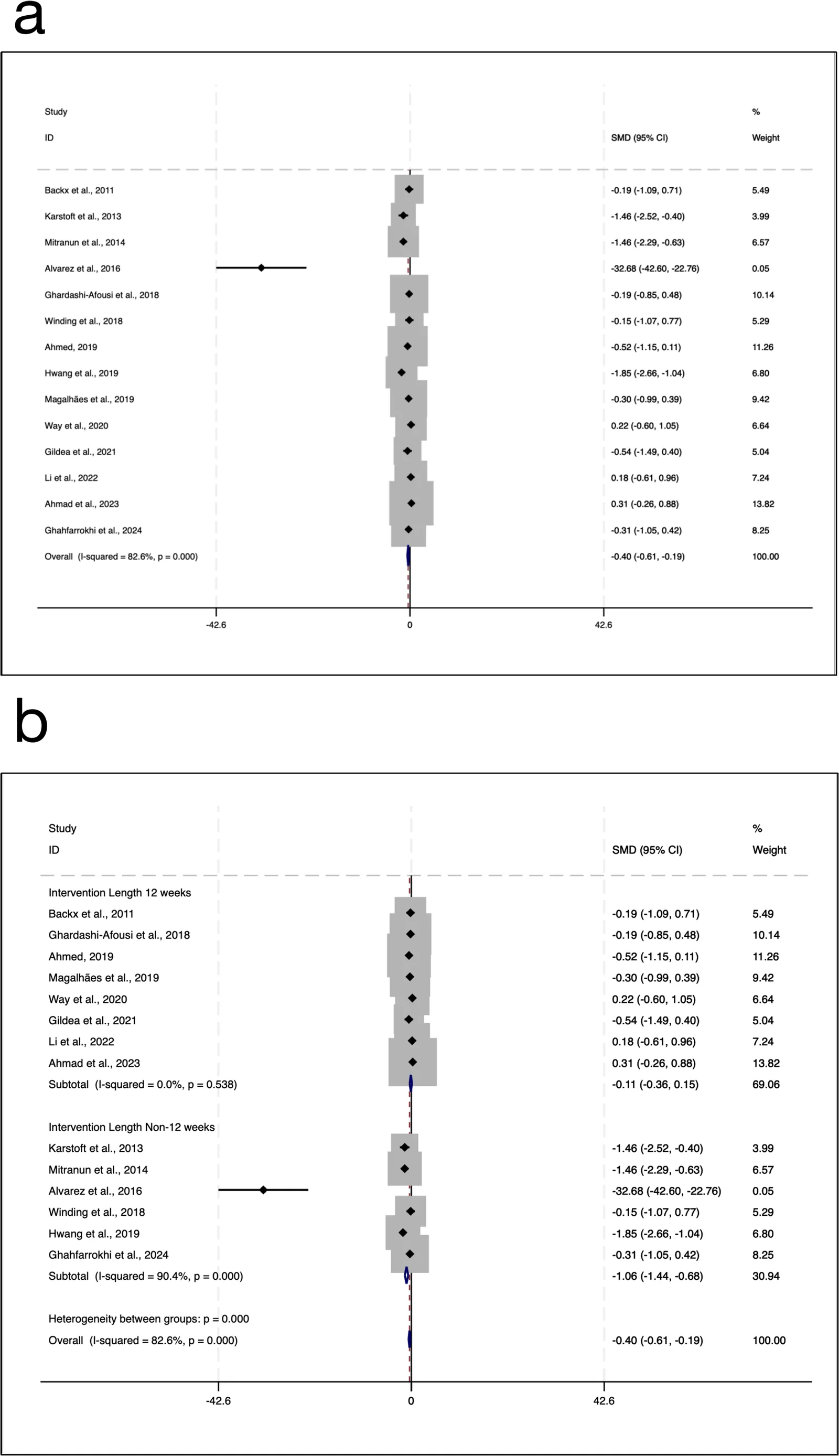
Effect of HIIT on BMI: **(a)** overall meta-analysis and **(b)** subgroup analysis by intervention duration (12 weeks vs non–12 weeks).

For BMI, a total of 14 RCTs reported BMI outcomes (**Figure 8a**). HIIT significantly reduced BMI compared with controls (SMD = −0.40, 95% CI −0.61 to −0.19, 95%PI -1.809 to 0.812), with substantial heterogeneity (I² = 82.6%, p < 0.001). In the duration subgroup analysis (**Figure 8b**), trials with 12-week interventions showed a non-significant BMI change (SMD = −0.11, 95% CI −0.36 to 0.15; I² = 0.0%), whereas trials with non–12-week interventions demonstrated a larger significant reduction (SMD = −1.06, 95% CI −1.44 to −0.68; I² = 90.4%). The between-subgroup difference was significant (p < 0.001), indicating that intervention duration is an important contributor to between-study differences in BMI. Subgroup analyses by age and weekly frequency did not meaningfully explain the observed heterogeneity.

### 3.4 Continuous meta-regression

Meta-regression analyses showed no significant moderator effects of mean age or intervention duration for SBP, DBP, and BMI (**Supplementary Table 2**). Duration was significantly associated with the HDL-C effect estimate, while age was significantly associated with the TG effect estimate, with the joint model explaining 52.7% of between-study heterogeneity for TG. For body weight, the duration coefficient was borderline but the joint model was not significant. Overall, these findings suggest that age and intervention duration may explain heterogeneity for selected lipid outcomes, particularly TG, but no consistent moderator effect was observed across all cardiometabolic outcomes. Because the analyses were exploratory, aggregate-level, and based on a small number of trials, they should not be interpreted as evidence that age independently modifies individual treatment effects.

### 3.5 Sensitivity analysis

**Figure 9:**
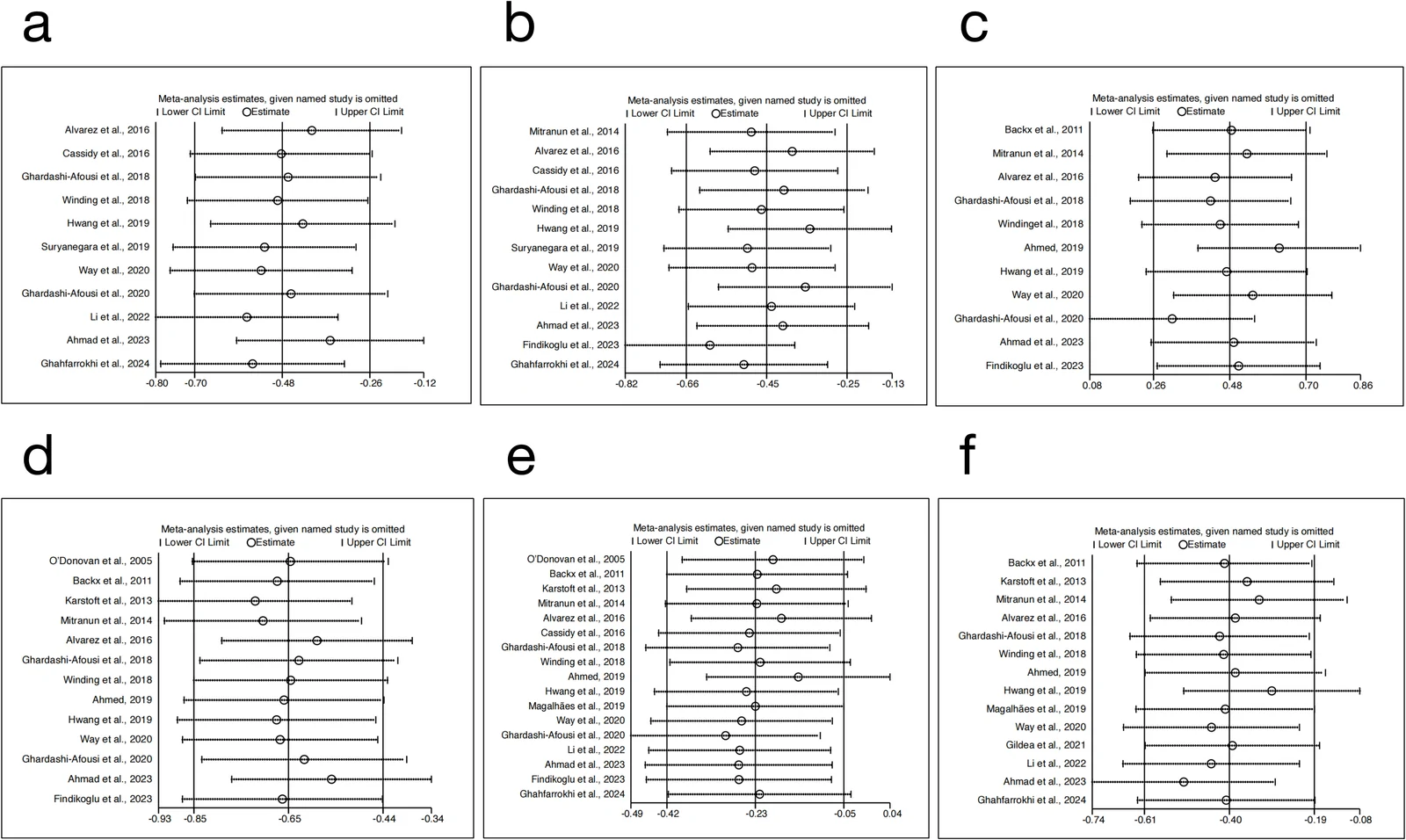
Leave-one-out sensitivity analyses for pooled outcomes: **(a–f)** SBP, DBP, HDL-C, TG, BW, and BMI.

Leave-one-out sensitivity analyses were performed for all six outcomes (**Figure 9**; panels a–f correspond to SBP, DBP, HDL-C, TG, BW, and BMI, respectively). Overall, sequential omission of individual trials yielded pooled estimates that remained broadly stable, and no single study exerted a disproportionate influence on the direction or magnitude of the summary effects. For SBP (panel a) and DBP (panel b), recalculated effect sizes clustered closely around the original pooled estimates, suggesting that the blood pressure findings were robust despite between-study heterogeneity. For HDL-C (panel c), the positive pooled effect was consistently preserved following the removal of each study, indicating that the observed increase in HDL-C was not driven by any single trial. Similarly, TG (panel d) showed a stable reduction with only modest fluctuations in effect size and confidence limits. For BW (panel e) and BMI (panel f), leave-one-out estimates demonstrated limited variability and retained the overall direction of effect. Collectively, these results indicate that the main conclusions of this meta-analysis are not materially affected by the inclusion of any individual RCT.

### 3.6 Tests for bias

**Figure 10:**
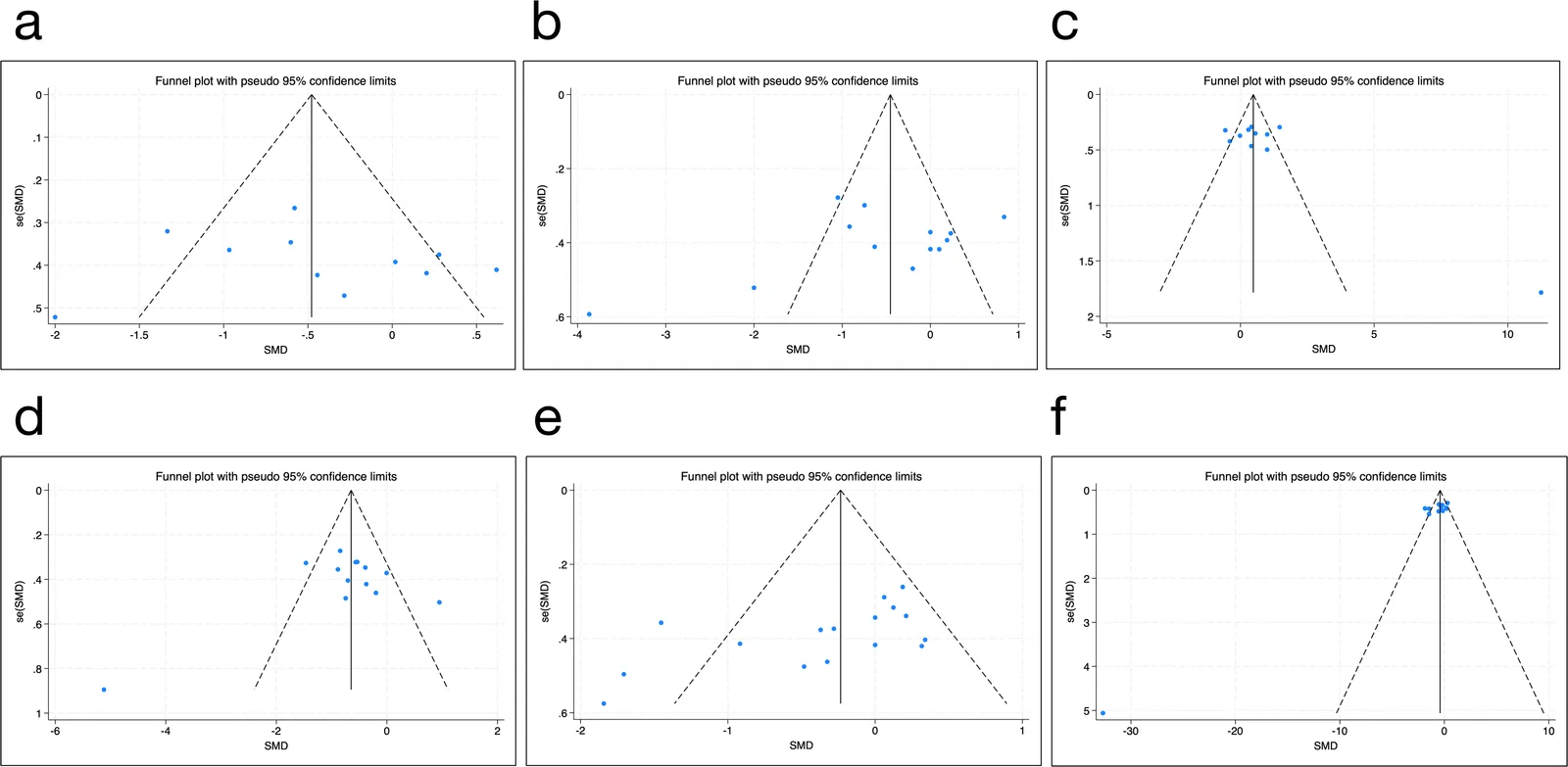
Funnel plots for assessment of small-study effects/publication bias: **(a–f)** SBP, DBP, HDL-C, TG, BW, and BMI.

By visual inspection of the funnel diagram with a pseudo-95% confidence interval of each outcome, the potential publication bias is evaluated (**Figure 10**; a to f corresponds to SBP, DBP, HDL-C, TG, BW and BMI respectively). In general, the funnel diagrams of SBP (panel a) and DBP (panel b) are generally symmetrically distributed on both sides of the combined effect value, but there is still a certain degree of discreteness, which is consistent with the greater heterogeneity observed in the meta-analysis. For HDL-C (panel c), the funnel diagram shows obvious asymmetry, which is mainly attributed to a prominent out-of-group study on the right, suggesting that there may be a significant impact of small-sample research effects and individual research characteristics. TG (panel d) shows mild asymmetry, and several studies are concentrated on one side of the combined effect value, indicating that the possibility of publication bias cannot be ruled out. BW’s funnel diagram (panel e) is relatively balanced. Each study is distributed on both sides of the combined effect value, and there is no obvious visual bias signal. In contrast, BMI (panel f) shows obvious asymmetry due to an extreme out-of-group value and the tight aggregation of the rest of the studies, which may reflect the effect of small-sample research, heterogeneity or selectivity reports. Overall, the funnel chart does not provide consistent evidence of significant publication bias in all outcomes; however, the asymmetry and out-of-group values shown by some endpoints, especially HDL-C and BMI, suggest that the results still need to be interpreted carefully.

Egger’s regression test did not indicate significant small-study effects for SBP, DBP, HDL-C, or TG, with p values of 0.726, 0.263, 0.070, and 0.437, respectively. However, significant small-study effects were detected for BW and BMI,with p values of 0.020 and 0.001 (**Supplementary Table 3**). Therefore, trim-and-fill analyses were further conducted for body weight and BMI. The trim-and-fill method imputed no potentially missing studies for either outcome, and the adjusted pooled effects were unchanged for BW, SMD = −0.23, 95% CI −0.42 to −0.05, and BMI, SMD = −0.40, 95% CI −0.88 to −0.12 (**Supplementary Table 4**).

### 3.7 Certainty of evidence

The certainty of evidence for all outcomes was assessed using the GRADE approach (**Supplementary Table 5**). Overall, the certainty of evidence was rated low for SBP and very low for DBP, HDL-C, TG, BW, and BMI. Downgrading was primarily attributable to serious risk of bias, serious inconsistency except SBP, and serious imprecision across the included studies. Publication bias was not detected for SBP, DBP, TG, and HDL-C, whereas publication bias was suspected for BW and BMI based on the overall assessment of publication bias. Indirectness was not considered serious for any outcome because the included studies were generally consistent with the review question regarding intervention and outcomes, although the eligible population included both overweight and obesity as defined by study authors.

## 4 Discussion

This systematic review and meta-analysis synthesizes 19 RCTs evidence published as of December 2025 to evaluate the effect of HIIT in adults with overweight or obesity. Included trials differed in exercise mode, interval structure, intensity prescription, supervision, intervention duration, participant cardiometabolic profile, and comparator content. Despite this clinical diversity, the pooled analyses suggested that HIIT was associated with improvements in several cardiometabolic risk indicators, including lower SBP, DBP, TG, BW, and BMI and higher HDL-C. However, heterogeneity was moderate to substantial for several outcomes, prediction intervals were wide for some endpoints, and GRADE certainty was low to very low. Exploratory meta-regression showed no significant moderator effects of mean age or intervention duration for SBP, DBP, and BMI, while study-level mean age was associated with TG effects and duration was associated with HDL-C effects. These findings support cautious, hypothesis-generating interpretation: trial-level mean age may be associated with differential responses for selected outcomes, but the available aggregate data do not prove age as an independent individual-level effect modifier.

### 4.1 Blood pressure (SBP and DBP)

A key finding of the revised analysis is that HIIT was associated with modest blood pressure reductions when SBP and DBP were re expressed as weighted mean differences in mmHg. This metric provides a more clinically interpretable estimate than the original standardized effect size. For SBP, HIIT was associated with a significant overall reduction, and significant reductions were observed in both participants younger than 55 years and those aged 55 years and older. However, the between subgroup difference was not statistically significant, indicating that the SBP response did not differ clearly by age group. For DBP, the overall effect was smaller and borderline, while the reduction was more evident among participants younger than 55 years and was not statistically significant among those aged 55 years and older. Nevertheless, the between subgroup difference for DBP did not reach statistical significance, suggesting that the apparent age-related pattern should be interpreted cautiously rather than as definitive evidence of age modification.

The observed pattern remains biologically plausible, but the strength of inference is limited. Obesity is closely associated with endothelial dysfunction, oxidative stress, chronic inflammation and impaired regulation of vascular tone, which together contribute to elevated blood pressure and increased cardiovascular risk (Banerjee and Mani, 2025). Obesity related vascular injury is increasingly understood as a systemic process involving adipose tissue inflammation and disruption of vascular homeostasis, and these mechanisms may interact with age related vascular remodeling and arterial stiffness (Liu and Yang, 2025). Mechanistic evidence further suggests that endothelial dysfunction is a central pathway linking obesity and hypertension, while chronic inflammation and perivascular adipose tissue dysfunction may aggravate impaired vascular regulation (Młynarska et al., 2025). These mechanisms may partly explain why the blood pressure response to HIIT varies across trials and participant profiles.

The weaker and more variable DBP response among older participants may reflect differences in baseline vascular phenotype, comorbidity burden and medication use rather than age alone. Older adults with obesity often present with greater arterial stiffness, more frequent multimorbidity and higher use of antihypertensive or metabolic medications, all of which may influence exercise induced hemodynamic adaptation and increase between study variability. Evidence from exercise interventions in older adults also indicates that blood pressure responses vary substantially according to participant characteristics, baseline risk and intervention delivery (Liang et al., 2024). In mixed sex samples, menopausal status may further contribute to variability because menopause is associated with adverse changes in body composition, insulin resistance, dyslipidemia and vascular function, which may influence cardiometabolic responsiveness in middle aged and older women (D’Costa et al., 2025). Therefore, the present subgroup findings should be viewed as exploratory signals rather than conclusive evidence that age independently determines the blood pressure response to HIIT.

Substantial heterogeneity remained, particularly for DBP. This heterogeneity may reflect differences in baseline blood pressure, antihypertensive medication use, blood pressure measurement procedures, adherence and the actual intensity achieved during HIIT. Current guidance on random effects meta-analysis emphasizes that heterogeneity is expected when complex interventions are synthesized across different populations and settings, and that the pooled estimate should be interpreted as an average effect within a distribution of possible effects rather than as a single universally applicable effect (McKenzie and Veroniki, 2024). From a practical perspective, the findings suggest that HIIT may contribute to modest blood pressure improvement in adults with obesity, but the magnitude and consistency of response may depend on participant characteristics and intervention implementation. Future trials should standardize the reporting of medication use, baseline blood pressure status, adherence, delivered exercise intensity and blood pressure measurement protocols to clarify which subgroups are most likely to benefit.

### 4.2 Lipid outcomes (HDL C and TG)

The results of the overall increase in HDL-C and the overall decrease in TG are consistent with the recent evidence that HIIT can improve the cardiometabolic health indicators of high-risk people, including patients with metabolic syndrome and diabesity. A recent RCT system review and meta-analysis report for adults with metabolic syndrome pointed out that HIIT has a positive effect on cardiometabolic health and supports HIIT as an effective exercise strategy, among which low-capacity training programs can also be a viable alternative to traditional aerobic exercise (Poon et al., 2024). Among the relevant population, a recent meta-analysis of patients with diabesity also evaluated a wide range of cardiometabolic outcomes and pointed out that the study conclusions may be affected by heterogeneity and insufficient evidence certainty, which further emphasizes the importance of carefully interpreting the results and improving the trial report (Al-Mhanna et al., 2025).

In this review, TG showed a clear overall decline, and the exploratory subgroup and meta-regression analyses suggested an association between study-level mean age and TG response. The decline appeared larger in trials with mean participant age <55 years, whereas the pooled effect in trials with mean participant age >=55 years was not statistically significant. This pattern is consistent with the hypothesis that aging-related changes in mitochondrial function, skeletal muscle oxidative capacity, insulin sensitivity, and obesity-related inflammation may influence lipid responses to HIIT (Wang et al., 2025). However, the evidence remains ecological because trial-level mean age may be confounded by sex distribution, baseline BMI, baseline TG, medication use, intervention duration, supervision, comparator content, and other design features. Therefore, the TG findings should be interpreted as a signal for future testing rather than as confirmation that age independently regulates metabolic adaptation to HIIT.

HDL-C increased overall, but the age interaction was not significant. Diet, weight change, lipid-lowering medication, fasting status, and laboratory methods may influence HDL-C estimates and may differ by age (Smart et al., 2025). These confounders were reported inconsistently, so differential effects in older and younger adults could not be tested. Future trials should report these variables in a standardized manner.

### 4.3 Anthropometric outcomes (BW and BMI)

Reductions in BW and BMI were small, consistent with the view that exercise alone often produces modest weight loss unless combined with dietary energy restriction or behavioral support. Cardiometabolic adaptations may still occur without large weight changes (Luo et al., 2025). Age did not modify BW, whereas intervention duration modified BMI; however, the non-12-week BMI subgroup remained highly heterogeneous. Dietary intake, baseline obesity severity, adherence, supervision, actual intensity, and total training volume may explain part of this variability.

These findings also highlight the limitations of using BW and BMI as outcome indicators. They cannot reflect fat distribution or body composition, which are key factors in the metabolic risk associated with obesity. The review of obesity-related vascular damage and systemic mechanisms emphasizes that adipose tissue dysfunction and vascular reconstruction are closely related to the risk of cardiometabolic, and these changes may be better reflected through indicators such as waist circumference, visceral fat and body composition, not just weight itself (Dai et al., 2023). Future experiments incorporating these measurement indicators may be able to more clearly clarify the effect of HIIT in improving the metabolic health of obese adults.

### 4.4 Subgroup findings and heterogeneity

The subgroup findings should be interpreted primarily as exploration of heterogeneity rather than as definitive evidence of treatment-effect modification. Age stratification appeared most informative for TG and less consistent for blood pressure, HDL-C, BW, and BMI. Weekly training frequency did not show consistent effect modification, while intervention duration had outcome-specific associations, particularly for HDL-C and BMI. Residual heterogeneity remained substantial within several subgroups, indicating that age, duration, and frequency alone do not adequately explain between-trial variability. The HIIT dose itself is multidimensional and depends on actual intensity, interval structure, exercise modality, session volume, progression, supervision, adherence, and background care; these details were often incompletely reported in the original trials. Similarly, comparator conditions varied and were often labeled as routine care or usual practice without sufficient detail about diet, medication, or non-HIIT physical activity. Recent exercise-intervention reporting guidance emphasizes the need to report FITT characteristics, individualized adjustment, adherence, adverse events, and control-group settings (Wollesen et al., 2025) (Subialka et al., 2025). Although this review includes adults with overweight or obesity rather than older adults alone, these reporting frameworks highlight a common challenge: incomplete intervention and comparator descriptions limit heterogeneity interpretation and clinical translation.

### 4.5 Methodological quality and implications for certainty

The GRADE assessment indicated low certainty for SBP and very low certainty for DBP, HDL-C, TG, BW, and BMI, suggesting that confidence in the pooled estimates remains limited despite several statistically significant findings. Downgrading was mainly driven by methodological limitations of the included trials, substantial between-study heterogeneity, and imprecision of the pooled estimates rather than by indirectness. Although publication bias was not detected for SBP, DBP, TG, and HDL-C by Egger testing, potential small-study effects could not be completely excluded. For BW and BMI, publication bias was considered more likely; however, trim-and-fill analyses did not identify missing studies and the pooled estimates remained unchanged after adjustment. Nevertheless, the low-to-very-low certainty of evidence indicates that future well-designed randomized controlled trials may substantially change the current estimates.

The exploratory meta-regression analyses provided limited evidence that study-level moderators explained heterogeneity across outcomes. Mean age and intervention duration did not consistently moderate the effects of HIIT on blood pressure or body composition outcomes. However, duration was associated with HDL-C effects, and mean age appeared to explain part of the heterogeneity in TG response. These findings may suggest that lipid responses to HIIT are more sensitive to participant age distribution and intervention exposure than blood pressure or anthropometric outcomes. Nevertheless, these results should be interpreted cautiously because meta-regression was conducted at the study level, the number of studies for each outcome was limited, residual heterogeneity remained for several outcomes, multiple moderator tests were performed, and individual participant data were unavailable. Future trials should report age distribution, sex proportion, baseline BMI, baseline cardiometabolic status, intervention duration, adherence, delivered intensity, supervision, comparator content, medication changes, and dietary co-interventions more consistently to allow more robust multivariable moderator analyses.

### 4.6 Sensitivity analysis, publication bias, and interpretive caution

The sensitivity analysis of the one-by-one elimination method supports the stability of the combined effect of the six outcomes, suggesting that the research conclusions are not dominated by any single experiment. However, leave-one-out robustness cannot rule out systematic bias across trials or resolve heterogeneity. For the research effect of publication bias and small samples, recent methodological literature reminds that it may be misleading to rely only on the appearance of the funnel diagram to make judgments, especially in the case of greater heterogeneity and large differences in research accuracy (Afonso et al., 2024). Publication bias was not strongly indicated for most outcomes based on Egger’s regression test. However, small-study effects were detected for body weight and BMI. Although trim-and-fill analysis did not impute missing studies and the adjusted estimates were unchanged, publication bias and small-study effects cannot be completely ruled out. Therefore, the certainty of evidence for body composition outcomes was downgraded and the findings should be interpreted cautiously.

### 4.7 Choice of effect size metric and clinical interpretability

Because SBP and DBP were measured in mmHg across the included trials, the revised analysis used weighted mean differences as the primary effect size metric for blood pressure outcomes. This approach improves clinical interpretability compared with an SMD-based synthesis because the pooled effects can be interpreted as absolute changes in SBP and DBP. Methodological guidance indicates that SMDs are appropriate when continuous outcomes are measured or reported on different scales, but their interpretation should be cautious because the standard deviation used for standardization affects the magnitude and comparability of the effect estimate (Gallardo‐Gómez et al., 2024). In contrast, when outcomes are reported on the same scale, mean differences are generally preferable because they preserve the original clinical unit (Jing and Lin, 2024). Accordingly, SBP and DBP were reanalyzed as WMDs in mmHg, enabling the findings to be interpreted against clinically meaningful blood pressure changes. Using an approximately 5-mmHg reduction as a conservative benchmark for clinically relevant blood pressure improvement, the revised results suggest that HIIT was associated with modest reductions in blood pressure, although average effects did not consistently reach a clearly clinically meaningful magnitude (Lin et al., 2025). For adults with overweight or obesity in trials with mean age <55 years, HIIT was associated with favorable changes in several cardiometabolic markers and may be considered as a time-efficient exercise option when clinically appropriate. For trials with mean age >=55 years, evidence was less precise and less consistent; HIIT may be considered individually after assessment of cardiovascular, musculoskeletal, metabolic, and functional status, with conservative progression and appropriate supervision. These subgroup findings should guide hypothesis generation rather than rigid age-based prescribing because they rely on study-level mean age and unadjusted multiple comparisons.

From a clinical point of view, these findings support HIIT as a potentially useful, time-efficient strategy for improving selected cardiometabolic risk markers in adults with overweight or obesity, but they do not justify a firm age-based prescription rule. This is consistent with the current public-health emphasis on increasing participation in moderate- and vigorous-intensity physical activity to improve health, as reflected in World Health Organization guidance on physical activity and sedentary behavior (World Health Organization, [[NoYear]]c). For older adults, recent global expert consensus emphasizes individualized, multicomponent exercise programs to promote healthy longevity and functional improvement, further supporting the need to tailor exercise prescription to health status rather than chronological age alone (Izquierdo et al., 2025). These views support the prioritization of safety, individualization and gradual dosage arrangements in practice, especially for older adults with obesity or high cardiometabolic burden.

Regarding safety, the recent review suggests that HIIT is feasible and safe under the premise of proper supervision, careful screening of participants, monitoring adverse events and taking conservative progress. A recent systematic review and meta-analysis report of mixed methods for stroke survivors pointed out that it is safe and feasible to implement HIIT on carefully screened individuals under supervised conditions. No deaths related to HIIT have been found, and there is no significant increase in adverse events compared with other exercise methods (Blatgé et al., 2025). Although this evidence is not specifically aimed at obese people, it supports a broader principle that high-intensity training programs can be safely implemented as long as risk screening and monitoring are appropriate. A recent narrative review also pointed out that HIIT has been safely used in many high-risk people, including patients with obesity and metabolic syndrome, and the incidence of adverse reactions reported in the literature is low, but it also points out that there are differences in the quality of monitoring and reporting in different studies (Reljic, 2025). These findings emphasize that future HIIT trials focusing on obese people should systematically report adverse events and risk screening procedures to enhance the clinical transformation value of the study results.

From the perspective of research, several priority directions can be put forward. First of all, future trials should improve the reporting of intervention doses in accordance with the structured reporting guidelines, including the actual intensity, interval structure, advancement, supervision, compliance and control conditions. Second, trials should measure and report common co-interventions and confounders, including dietary intake, medication changes, baseline physical activity, baseline BMI, sex distribution, and baseline cardiometabolic status, and should standardize outcome measurement protocols. Third, future studies should incorporate more sensitive adiposity outcomes, such as waist circumference, visceral fat, and body composition, to better capture clinically meaningful changes. Finally, adequately powered RCTs with individual-participant age data and prespecified age-by-treatment interaction analyses are needed to test whether age modifies cardiometabolic response to HIIT and to identify optimal HIIT dosing for clinically relevant subgroups.

### 4.8 Strengths and limitations

Strengths: This review only includes RCT of obese adults, and examines a group of clinically relevant and relatively consistent outcome indicators, including SBP, DBP, HDL-C, TG, BW and BMI. The study improved clinical interpretability by reporting SBP and DBP as weighted mean differences in mmHg, incorporated prediction intervals and GRADE certainty assessment, explored heterogeneity using prespecified subgroup analyses and continuous study-level meta-regression, and interpreted age-related findings cautiously as aggregate-level signals.

Limitations: Several limitations directly affect interpretation. First, age analyses were based on study-level mean age rather than individual-participant data; therefore, they are vulnerable to ecological bias and confounding by trial-level differences such as sex distribution, baseline BMI, baseline cardiometabolic status, medication use, dietary advice, intervention modality, supervision, duration, and comparator content. The 55-year threshold was pragmatic and partly motivated by menopause-related cardiometabolic changes, a rationale that applies most directly to female-predominant samples and less clearly to mixed-sex trials. Second, heterogeneity remained moderate to substantial for several outcomes and often persisted within subgroups, indicating that age, duration, and frequency did not fully explain between-study variability. Third, although SBP and DBP were reanalyzed as WMDs in mmHg, lipid and anthropometric outcomes were synthesized using SMDs, which reduces direct clinical interpretability. Fourth, the primary random-effects analyses used the DerSimonian-Laird estimator; this conventional approach may provide overly narrow uncertainty estimates in small meta-analyses with substantial heterogeneity, so the findings were interpreted conservatively alongside prediction intervals, sensitivity analyses, and GRADE certainty. Fifth, the eligibility criteria allowed adults with overweight or obesity as defined by study authors, creating a broader population than an exclusively obesity-defined sample. Sixth, comparator conditions were described variably as standard care, usual practice, or routine care and were often insufficiently detailed with respect to background exercise, diet, medication, or clinical follow-up. Finally, important outcomes such as waist circumference, body composition, visceral adiposity, glycemic markers, adverse events, and longer-term follow-up were not consistently reported for quantitative synthesis.

## 5 Conclusion

This systematic review and meta-analysis incorporates 19 RCTs published as of December 2025. The results show that HIIT can improve many cardiometabolic risk indicators among adults with overweight or obesity, including reductions in SBP, DBP, TG, BW, and BMI and an increase in HDL-C. However, heterogeneity was substantial for several outcomes, prediction intervals were wide for some endpoints, and certainty of evidence was low for SBP and very low for the other outcomes. Exploratory meta-regression showed no significant moderator effects of mean age or intervention duration for SBP, DBP, and BMI, while study-level mean age was associated with TG effects and intervention duration was associated with HDL-C effects. These findings should be interpreted as aggregate-level associations rather than proof of individual-level age effect modification.

For adults with overweight or obesity, HIIT may be considered as a time-efficient exercise strategy when clinically appropriate, particularly when screening, supervision, gradual progression, and safety monitoring are available. The apparent differences between trials with mean age <55 years and >=55 years should be used for hypothesis generation and future trial design rather than for rigid age-based prescribing. The 55-year threshold is pragmatic, not a universal biological or clinical boundary.

Future RCTs should recruit larger and more diverse samples, prespecify adequately powered individual-level age analyses, standardize reporting of intervention dose and comparator care, record adherence and adverse events systematically, measure and report medication, diet, baseline physical activity, sex distribution, baseline BMI, and baseline cardiometabolic status, extend follow-up, and include more sensitive adiposity and metabolic outcomes. Future evidence syntheses should apply contemporary risk-of-bias and certainty frameworks, consider robust random-effects inference when data permit, and use individual-participant or richer study-level data to improve clinical interpretability and investigate heterogeneity.

## Data Availability

All publicly available datasets used in this study can be found at: PubMed (https://pubmed.ncbi.nlm.nih.gov/), Web of Science (https://webofscience.clarivate.cn/), Embase (https://www.embase.com/landing?status=grey), and Cochrane Library (https://www.cochranelibrary.com/).
