# Supplementary material for: Study-level age patterns in cardiometabolic response to high-intensity interval training among adults with overweight or obesity: a systematic review and meta-analysis of randomized controlled trials
Source: Front Physiol. 2026 Aug 12;17:1833719. doi: 10.3389/fphys.2026.1833719 (PMC13506465; doi:10.3389/fphys.2026.1833719)
Supplement: Supplementary file 1 [file Table1.docx]

**Supplemental Table 1.** Characteristics of the included RCTs.

| **Study** |  | | | | **Participants characteristics** | | **Intervention characteristics** | | | **Control group** | **Outcome** |
| --- | --- | --- | --- | --- | --- | --- | --- | --- | --- | --- | --- |
|  | **Region** | **Study design** | **obesity definition** | **Age** | | **Sample Size (N)** | **Intervention Description** | **Intervention Length (Weeks)** | **Frequency** |  |  |
| O’Donovan et al., 2005 | United Kingdom | RCT | Type 2 diabetes | 41.5±1.5 | | I: 13 C: 13 | 3x/week; work intervals: 70%−80% VO2max; cycling (supervised, field-based) | 24 | 3 | Routine Prenatal care | TG, BW |
| Backx et al., 2011 | United Kingdom | RCT | Type 2 diabetes | 59.6±12.5 | | I: 10 C: 9 | 3x/week.; work intervals:  5–10×1–3 min (80%−90% HRR), rest intervals: 5–10×1–2 min (40%−50% HRR); 20–40 min; cycling (supervised, lab-based) | 12 | 3 | Routine Prenatal care | HDL-C, TG, BW |
| Karstoft et al., 2013 | Denmark | RCT | Type 2 diabetes | 60.8±2.2 | | I: 10 C: 8 | 5x/week, work intervals: 10×3 min  (70% HRmax), rest intervals: 10×3 min  (60% HRmax); 60 min; walking (unsu_x0002_pervised, field-based) | 16 | 5 | Routine Prenatal care | TG, BW, BMI |
| Mitranun et al., 2014 | Thailand | RCT | Type 2 diabetes | 60.9±2.4 | | I: 14 C: 15 | 3x/week.; work intervals: 4–6×1 min  (80%−85% VO2peak), rest intervals: 3–5×4 min (50%−60% VO2peak);  16–26 min; jogging/walking (super_x0002_vised, lab-based) | 12 | 5 | Routine Prenatal care | DBP, HDL-C, TG, BW, BMI |
| Alvarez et al., 2016 | Chile | RCT | Type 2 diabetes | 44.5±1.8 | | I: 13 C: 10 | 3x/week.; work intervals:  8–14×30–60 s (90–100% HRR, RPE  15–17, jogging/running), rest intervals: 9–15×90–120 s (≤70%, RPE<9, walk_x0002_ing), 3 min (50/50 active/passive ratio); 22–37.5 min; cycling (supervised,  lab-based) | 16 | 3 | Routine Prenatal care | SBP, DBP, HDL-C, TG, BW, BMI |
| Cassidy et al., 2016 | United Kingdom | RCT | Type 2 diabetes | 61.0±9.0 | | I: 12 C: 11 | 3x/week.; work intervals: 5×2–4 min  (RPE 16–17), rest intervals: 4×3 min  (50/50 active/passive ratio);  22–31 min; cycling (unsupervised,  field-based) | 12 | 3 | Routine Prenatal care | SBP, DBP, BW |
| Ghardashi-Afousi et al., 2018 | Iran | RCT | Type 2 diabetes | 54.0±8.0 | | I: 18 C: 17 | 3x/week; work intervals: 12×1.5 min  (85%−90% HRmax; rest intervals:  11×2 min (55%−60% HRmax); 40 min;  cycling (supervised, lab-based) | 12 | 3 | Routine Prenatal care | SBP, DBP, HDL-C, TG, BW, BMI |
| Winding et al., 2018 | Denmark | RCT | Type 2 diabetes | 57.0±7.0 | | I: 13 C: 7 | 3x/week.; work intervals: 10×1 min  (95% HRpeak), rest intervals: 10×1 min  (25% HRpeak); 20 min; cycling (super_x0002_vised, lab-based) | 11 | 3 | Routine Prenatal care | SBP, DBP, HDL-C, TG, BW, BMI |
| Ahmed, 2019 | Egypt | RCT | Type 2 diabetes | 52.4±4.6 | | I: 20 C: 20 | 3x/week.; work intervals: 2×4 min  (85%−90% HRpeak), rest intervals:  1×3 min (65%−75% HRpeak);  11 min; jogging/walking (supervised,  lab-based) | 12 | 3 | Routine Prenatal care | HDL-C, TG, BW, BMI |
| Hwang et al., 2019 | United States | RCT | Type 2 diabetes | 65.0±2.0 | | I: 18 C: 16 | 4x/week.; work intervals: 4×4 min  (90% HRpeak), rest intervals: 3×3 min  (70% HRpeak); 25 min; cycling (super_x0002_vised, lab-based) | 8 | 4 | Routine Prenatal care | SBP, DBP, HDL-C, TG, BW, BMI |
| Suryanegara et al., 2019 | United Kingdom | RCT | Type 2 diabetes | 60.5±8.6 | | I: 13 C: 13 | 3x/week.; work intervals: 5×2–4 min  (RPE 16–17), rest intervals: 4×3 min  (50/50 active/passive ratio);  22–31 min; cycling (unsupervised,  field-based) | 12 | 3 | Routine Prenatal care | SBP, DBP |
| Magalhães et al., 2019 | Portugal | RCT | Type 2 diabetes | 59.0±8.1 | | I: 13 C: 22 | 3x/week.; work intervals:10×1–2 min  (70%−90% HRR), rest intervals:  10×1 min (40%−60% HRR); 33 min;  cycling (supervised, lab-based) | 12 | 3 | Routine Prenatal care | BW, BMI |
| Way et al., 2020 | Australia | RCT | Type 2 diabetes | 56.9±2.1 | | I: 12 C: 11 | 3x/week.; work intervals: 1×4 min  (90% VO2peak); 4 min; cycling (super_x0002_vised, lab-based) | 12 | 3 | Routine Prenatal care | SBP, DBP, HDL-C, TG, BW, BMI |
| Ghardashi-Afousi et al., 2020 | Iran | RCT | Type 2 diabetes | 54.6±5.9 | | I: 30 C: 29 | 3x/week.; work intervals: 6×4 min  (85%−90% HRmax), rest intervals:  5×3 min (50/50 active/passive ratio);  39 min; cycling (supervised, lab-based) | 12 | 3 | Routine Prenatal care | SBP, DBP, HDL-C, TG, BW |
| Gildea et al., 2021 | United Kingdom | RCT | Type 2 diabetes | 53.0±10.0 | | I: 9 C: 9 | 3 days/week; work intervals: 10×1 min  (~90% HRmax), rest intervals: 9×1 min  (active); 19 min; cycling (supervised,  lab-based) | 12 | 3 | Routine Prenatal care | BMI |
| Li et al., 2022 | China | RCT | Type 2 diabetes | 39.0±5.0 | | I: 13 C: 12 | 5x/week; work intervals: 8×1 min  (80%−95% HRmax), rest intervals:  7×1 min (20%−30% HRmax, active or  passive); 15 min; cycling (supervised,  lab-based) | 12 | 3 | Routine Prenatal care | SBP, DBP, BW, BMI |
| Ahmad et al., 2023 | Egypt | RCT | Type 2 diabetes | 43.3±6.2 | | I: 24 C: 24 | 3x/week; work intervals: 4×4 min  (80%−90% HRmax), 4×3 min  (50%−60% HRmax); 28 min; jogging/ walking (supervised, lab-based) | 12 | 3 | Routine Prenatal care | SBP, DBP, HDL-C, TG, BW, BMI |
| Findikoglu et al., 2023 | Turkey | RCT | Type 2 diabetes | 57.5±7.8 | | I: 20 C: 20 | 3x/week; work intervals: 8–16×1 min  (90% VO2peak), 7–15×2 min (30% VO- 2peak); 24–48 min; cycling (supervised,  lab-based) | 12 | 3 | Routine Prenatal care | DBP, HDL-C, TG, BW |
| Ghahfarrokhi et al., 2024 | Iran | RCT | Type 2 diabetes | 67.5±5.8 | | I: 16 C: 13 | 3x/week; 10–14 repetitions/exercise  (75%–85% HRR); 1:1 work/rest ratio; 30–35 min; multicomponent function_x0002_al training (supervised, field-based) | 6 | 3 | Routine Prenatal care | SBP, DBP, BW, BMI |

Abbreviations:I: Intervention; C: Control; SBP (control; systolic blood pressure); DBP (diastolic blood pressure); HDL-C (high-density lipoprotein cholesterol); TG (triglyceride); BW (Body Weight); and BMI (Body Mass Index).

**Supplemental Table 2.** Continuous meta-regression analysis

| Outcome | Age β (p) | Duration β (p) | Joint model |
| --- | --- | --- | --- |
| SBP | 0.0194 (0.419) | -0.1183 (0.164) | p=0.408; R²=0.0% |
| DBP | 0.0184 (0.571) | 0.0131 (0.916) | p=0.731; R²=0.0% |
| HDL-C | -0.0995 (0.074) | 0.5919 (0.009) | p=0.028; R²=0.0% |
| TG | 0.0798 (0.003) | -0.0275 (0.648) | p=<0.001; R²=52.7% |
| BW | 0.0064 (0.743) | -0.0744 (0.067) | p=0.122; R²=11.8% |
| BMI | -0.0309 (0.359) | -0.0868 (0.489) | p=0.371; R²=0.6% |

**Supplemental Table 3.** Egger Test of all outcomes.

| Outcome | 95%CI | P |
| --- | --- | --- |
| SBP | -6.101327, 8.422536 | 0.726 |
| DBP | -11.88893, 3.595093 | 0.263 |
| HDL-C | -.4797057, 10.1979 | 0.07 |
| TG | -7.120304, 3.299696 | 0.437 |
| BW | -8.634343, -.8715702 | 0.02 |
| BMI | -9.145957, -3.013726 | 0.001 |

**Supplemental Table 4.** Trim-and-fill analysis for BW and BMI.

| Outcome | Observed studies | Imputed studies | Original SMD, 95% CI | Adjusted SMD, 95% CI |
| --- | --- | --- | --- | --- |
| BMI | 14 | 0 | −0.40, −0.88 to −0.12 | −0.50, −0.88 to −0.12 |
| BW | 17 | 0 | −0.23, −0.42 to −0.05 | −0.23, −0.42 to −0.05 |

**Supplemental Table 5.** GRADE assessment of the certainty of evidence for each outcome.

| Outcome | Risk of bias | Inconsistency | Indirectness | Imprecision | Publication bias | Certainty |
| --- | --- | --- | --- | --- | --- | --- |
| SBP | Serious | Not serious | Not serious | Serious | Undetected | Low |
| DBP | Serious | Serious | Not serious | Serious | Undetected | Very low |
| HDL-C | Serious | Serious | Not serious | Serious | Undetected or Potential concern | Very low |
| TG | Serious | Serious | Not serious | Serious | Undetected | Very low |
| BW | Serious | Serious | Not serious | Serious | Suspected | Very low |
| BMI | Serious | Serious | Not serious | Serious | Suspected | Very low |
